# Polyhexamethylene Biguanide Nanoparticles Inhibit Biofilm Formation by Mastitis-Causing *Staphylococcus aureus*

**DOI:** 10.3390/vetsci12050507

**Published:** 2025-05-21

**Authors:** Renata de Freitas Leite, Breno Luis Nery Garcia, Kristian da Silva Barbosa, Thatiane Mendes Mitsunaga, Carlos Eduardo Fidelis, Bruna Juliana Moreira Dias, Renata Rank de Miranda, Valtencir Zucolotto, Liam Good, Marcos Veiga dos Santos

**Affiliations:** 1 Qualileite Milk Quality Laboratory, Department of Animal Nutrition and Production, School of Veterinary Medicine and Animal Science, University of São Paulo, Pirassununga 13635-900, Brazil; renata.frleite@gmail.com (R.d.F.L.); breno.luis.garcia@usp.br (B.L.N.G.); barbosa.kristian@usp.br (K.d.S.B.); tathiane.mitsunaga@usp.br (T.M.M.); carlosfidelis@usp.br (C.E.F.); 2 Nanomedicine and Nanotoxicology Group, Physics Institute of São Carlos, University of São Paulo, São Carlos 13560-970, Brazil; bruna.moreira@usp.br (B.J.M.D.); renatam.bio@gmail.com (R.R.d.M.); zuco@ifsc.usp.br (V.Z.); 3 Department of Pathobiology and Population Sciences, The Royal Veterinary College, University of London, London MW1 0TU, UK; lgood@rvc.ac.uk

**Keywords:** antimicrobial resistance, bovine mastitis, nanotechnology, intramammary treatment, teat disinfection, one health

## Abstract

*Staphylococcus aureus* is a mastitis pathogen that compromises cow health and causes significant economic losses in the dairy industry. High antimicrobial resistance and biofilm formation by *S. aureus* limit the efficacy of conventional treatments. This study evaluated the potential of polyhexamethylene biguanide nanoparticles (PHMB NPs) against mastitis-causing *S. aureus*. PHMB NPs showed low toxicity to bovine mammary epithelial cells (MAC-T cells) at concentrations up to four times higher than the minimum inhibitory concentration (1 µg/mL) against *S. aureus*. In Experiment 1, PHMB NPs significantly reduced biofilm formation by *S. aureus* by 50% at concentrations ≥1 µg/mL, though they showed limited efficacy against preformed biofilms. In Experiment 2, using an excised teat model, PHMB NPs reduced *S. aureus* concentrations by 37.57% compared to conventional disinfectants (chlorhexidine gluconate, povidone–iodine, and sodium dichloroisocyanurate), though limited by short contact time. These findings highlight the potential of PHMB NPs for the control of *S. aureus* growth and biofilm formation.

## Simple Summary

This manuscript addresses the persistent challenge of *Staphylococcus aureus*, a primary agent of bovine mastitis that compromises animal welfare and results in substantial economic losses in dairy production. The difficulty in treating *S. aureus* lies in its resistance to multiple antibiotics and its capacity to form resilient biofilms within mammary gland cells, which hampers the effectiveness of conventional therapies. In response, the study evaluates a novel strategy involving polyhexamethylene biguanide (PHMB) nanoparticles, known for their antimicrobial properties. Two main experiments were conducted. The first assessed the cytotoxicity of PHMB nanoparticles and their ability to disrupt *S. aureus* biofilms in vitro. The findings reveal that these nanoparticles were well tolerated by bovine mammary cells and significantly inhibited biofilm formation at effective concentrations. The second experiment compared PHMB nanoparticles to commonly used post-milking disinfectants. While the nanoparticles did not outperform traditional treatments, they still reduced bacterial load, with results possibly influenced by limited exposure time during application. Despite this, the study highlights the potential of PHMB nanoparticles as an alternative approach for controlling mastitis caused by *S. aureus*, especially in biofilm-associated infections. This innovative method may contribute to improved udder health and enhanced productivity in the dairy industry, warranting further investigation under practical conditions.

## 1. Introduction

Mastitis is an inflammatory reaction of the cow’s mammary gland (MG) triggered by a bacterial infection. Mastitis is the foremost endemic disease in dairy herds, compromising cows’ welfare and causing huge economic impacts on the dairy industry [1]. The economic losses are associated with the reduction in milk quality and production, discarded milk, culling, and costs related to veterinary assistance, diagnosis, and treatment [2].

*Staphylococcus aureus* is a major etiological pathogen associated with clinical and subclinical mastitis, and its prevalence ranges from 2 to 70.3% in dairy herds worldwide [3,4] Bacteriological cure rates of mammary quarters infected by *S. aureus* may achieve 44.7 and 37.7% for clinical and subclinical cases, respectively; however, spontaneous bacteriological cure is rare [5]. In addition to therapy failures, *S. aureus* is also a concern for public health since some *S. aureus* strains can contaminate and compromise the quality and safety of dairy products [6].

For these reasons, strategies to prevent mastitis caused by *S. aureus* are crucial and aim to prevent new cases of intramammary infections (IMI) in healthy cows and reduce the infection duration in already infected cows [7]. As *S. aureus* is usually transmitted from infected to healthy cows during milking, control programs against *S. aureus* include post-milking teat disinfection and segregation [8]. This practice is considered one of the most effective techniques to prevent IMI caused by *S. aureus* as it reduces the bacterial load on teat skin [9].

However, as the teat canal remains dilated for up to 2 h after milking [10], mammary quarters are prone to bacterial invasion [11]. The colonization of MG by *S. aureus* depends on the three main mechanisms: (i) adhesion to the host cell, (ii) invasion into tissues and cells, and (iii) evasion of the host immune system [12]. Consequently, intracellular bacteria are protected against host cell mechanisms and antimicrobials, persisting in a semi-dormant state, which results in antimicrobial resistance (AMR) [13].

The biofilm-forming ability of *S. aureus* may be associated with AMR, chronicity of IMI, higher antimicrobial use, and therapy failures [14]. Biofilms are formed after bacterial attachment to an inert or living surface, and then, the bacterial community self-produces an extracellular matrix composed of DNA, proteins, and polysaccharides. The main constituent of the matrix produced by *S. aureus* is the surface polysaccharide intercellular adhesin [15]. Bacteria inside the biofilm matrix present physiological changes associated with the biofilm microenvironment that may induce AMR. Moreover, the restricted penetration of antimicrobials across the biofilm matrix is another hypothesis that explains their lower susceptibility to antimicrobials [16]. Considering the biofilm formation inside the MG, *S. aureus* clusters were observed within the alveoli and lactiferous ducts of experimentally infected cows by microscopy [17]. In addition, the content of swabs taken from the teat cistern, gland cistern, and parenchyma of infected cows was evaluated by immunofluorescence staining, and polysaccharide intercellular adhesin, which is the main component of *S. aureus* biofilm matrix, was detected [18]. For these reasons, the development of affordable and novel materials capable of preventing MG invasion by *S. aureus* and its biofilm formation is essential for enhancing MG health and preventing AMR.

The formulation of new and existing compounds at the nanoscale level has been considered a potential strategy to prevent, treat, and overcome the challenges related to bovine mastitis [19]. Nanoparticles (NPs) are more reactive than their bulk analogs due to their size, spatial confinement, larger surface area and surface-to-volume ratio, higher level of energy, and proportion of surface atoms [20,21]. Promising results have been obtained regarding NP stability and usage for targeted drug delivery and controlled release, as well as NPs’ ability to cross biological barriers [22]. Furthermore, NPs excel at inhibiting pathogen growth and acting against biofilms. NPs’ antimicrobial activity is related to their ability to penetrate the cell membrane of pathogens and interfere with important molecular pathways, being able to act even against intracellular pathogens [23]. Finally, NPs’ antibiofilm activity occurs because of their size and shape, which enables their penetration into biofilm structures and prevention of biofilm formation through changes in adhesion forces [16,24].

Polymeric nanomaterials are synthesized using natural or synthetic compounds. The biomedical field has widely studied them as they are biocompatible, biodegradable, safe, and stable during storage. In addition, polymeric NPs present high antimicrobial and antibiofilm activities. Polyhexamethylene biguanide (PHMB) is a cationic and versatile polymer, which presents a broad spectrum of antimicrobial activity, including against mastitis pathogens such as *S. aureus* and *Prototheca* spp. [14,25]. Two mechanisms of action are involved in PHMB antimicrobial activity: microbial membrane disruption [26] and the ability to bind and condense bacterial DNA, which may not lead to resistance [27].

Previously, our group reported the high antimicrobial activity of PHMB NPs against mastitis-causing *S. aureus* and their stability during storage [28]. Here, we aimed to explore the potential applications of PHMB NPs against *S. aureus*. Therefore, in addition to PHMB NPs and *S. aureus* characterization, two major experiments were performed. Considering that *S. aureus* may form biofilms inside mammary tissue, the objective of Experiment 1 was to evaluate the toxicity of PHMB NPs to bovine mammary epithelial cells (MAC-T cells) and their antibiofilm activities. On the other hand, Experiment 2 aimed to test the potential use of PHMB NPs for teat disinfection, using the excised teat model to evaluate PHMB NPs’ ability to reduce *S. aureus* load on teat skin.

## 2. Materials and Methods

The experimental protocols were designed in accordance with the Ethical Principles in Animal Research adopted by the Ethics Committee for the Use of Animals of the School of Veterinary Medicine and Animal Science, University of São Paulo, Brazil.

### 2.1. Synthesis of Polyhexamethylene Biguanide Nanoparticles

The PHMB NPs were prepared in accordance with our previous study [28]. Briefly, 5 mL of ultrapure water and 2.5 mL of 1 mg/mL PHMB (kindly provided by Polyorganic Tecnologia-osé Diniz, 3651-Campo Belo, São Paulo, Brazil) were added to a polypropylene tube, followed by 3.75 mL of 1 mg/mL sodium alginate (Sigma-Aldrich, Oakville, ON, Canada) and a polytetrafluoroethylene stirring bar. The mixture was stirred using a magnetic mixer (Cole-Parmer, Vernon Hills, IL, USA) at 650 rpm for 20 min and then rested for 20 min at room temperature. Next, 3.75 mL of a low-molecular-weight chitosan (Sigma-Aldrich) solution in 1% acetic acid was added to the tube, and the mixture was stirred as previously reported [28], resulting in a final concentration of 167 µg/mL PHMB. After the synthesis procedures, 1 mL of PHMB NPs was transferred into a cuvette to characterize the particle population by dynamic light scattering (DLS) and zeta potential measurements on a Malvern Zetasizer Nano-ZS 90 (Malvern Instruments Ltd., Malvern, UK). As mentioned in our previous study [28], polymer-based nanoparticles (NPs) are colloidal structures less than 1000 nm in size, consisting of a polymeric core capable of encapsulating active substances [29].

### 2.2. Selection and Identification of Staphylococcus aureus Isolates

A total of 10 *S. aureus* isolates were selected from the collection of cryopreserved mastitis pathogens from the Milk Quality Research Laboratory (Qualileite), University of São Paulo (USP), Brazil. These isolates were selected based on an analysis of antimicrobial susceptibility conducted in previous studies by our research group [30,31]. Both studies were approved by the Ethics Committee on the Use of Animals of the School of Veterinary Medicine and Animal Sciences/USP. The most resistant isolates were intentionally selected. However, as these studies evaluated different antimicrobials, MIC assays were repeated with a higher number of antimicrobials and antimicrobial classes, aiming to standardize this information. Further, considering that there is usually one predominant *S. aureus* strain in each dairy herd, we established that the 10 isolates must have been collected from 10 different dairy herds. No further information was associated with their selection. To enhance variability, *S. aureus* isolates were obtained from individual milk samples collected from cows with clinical (n = 5) and subclinical (n = 5) mastitis from 10 commercial dairy herds located in southeast Brazil. Isolates were identified by microbiological procedures and MALDI-TOF MS [32] using the Flex Control 3.4 software and MBT Compass 4.1.7 software (Bruker Daltonik Inc., Bremen, Germany). Scores of ≥2 were considered reliable for colony identification at the species level. Following the numeric sequence of pathogen identification established by Qualileite, selected isolates were enumerated as 1650, 1651, 1658, 1663, 1666, 1667, 1677, 1680, 1684, and 1688.

### 2.3. Antimicrobial Susceptibility

The susceptibility of *S. aureus* (n = 10) to antimicrobials was evaluated using the broth microdilution assays to determine the minimum inhibitory concentrations (MICs) according to the European Committee on Antimicrobial Susceptibility Testing (EUCAST) guidelines [33]. Antimicrobials from 7 selected antimicrobial classes (aminoglycosides, cephalosporins, fluoroquinolones, lincosamide, macrolides, penicillin, and tetracyclines) were tested. This selection was based on the results of a study performed by our research group that characterized the treatment profile and quantified antimicrobial consumption for mastitis treatment in Brazilian dairy herds [34].

Amoxicillin, ampicillin, penicillin G, cefalexin, cefoxitin, ceftiofur, ciprofloxacin, enrofloxacin, erythromycin, gentamicin, lincomycin, oxytetracycline, and tetracycline were purchased from Sigma-Aldrich, while ciprofloxacin was purchased from InLab (InLab, São Luis, Brazil). Antimicrobials were freshly prepared in stock solutions at 1 mg/mL in accordance with the manufacturer’s instructions and serially diluted two-fold across the columns of a 96-well microplate (Cral), as previously reported [35]. A range of concentrations from 64 to 0.03 µg/mL of each antimicrobial was obtained in a final volume of 125 µL of Mueller–Hinton broth (MHB; Becton, Dickinson, and Co., Franklin Lakes, NJ, USA). Cryopreserved *S. aureus* isolates and the quality control strain *S. aureus* ATCC 29213 were recovered on blood agar and standardized at 5 × 10^6^ cfu/mL in 0.9% saline solution; afterward, 12.5 µL of bacterial suspension was added to each well, excluding the negative control. Plates were aerobically incubated at 37 °C, and MICs required to inhibit the growth of bacteria were determined by visual inspection after 24 h of incubation.

*S. aureus* isolates were classified as susceptible (S) or resistant (R) to antimicrobials in accordance with EUCAST breakpoints for this bacterial species [36]. Overall, isolates were considered resistant to penicillin (amoxicillin, ampicillin, and penicillin G) when the MIC was >0.125 µg/mL, which is the breakpoint established for benzylpenicillin. Resistance to cephalosporins (cefalexin, cefoxitin, and ceftiofur) was determined at the breakpoint of >4 µg/mL established for cefoxitin. Isolates were resistant to oxytetracycline at the same breakpoint used for tetracycline (>2 µg/mL), and the breakpoint (>1 µg/mL) established for ciprofloxacin was used for enrofloxacin. For lincomycin, we adapted the breakpoint of >0.5 µg/mL, which is used to classify *S. aureus* as resistant to clindamycin. Finally, isolates were resistant to erythromycin and gentamycin when the MIC was >2 µg/mL and >1 µg/mL, respectively. Furthermore, isolates that presented resistance to 3 or more antimicrobial classes were considered multi-resistant, and resistance to cefoxitin was considered an indicator of methicillin resistance (MRSA).

### 2.4. Intracellular Infection of MAC-T Cells

Five *S. aureus* isolates (1650, 1651, 1666, 1677, and 1684) were selected for the infection of MAC-T cells by gentamicin protection assays due to their strong ability to form biofilms, and the *S. aureus* strain 15 AL was used as an invasive reference [14]. Bacteria were cultured overnight in MHB (Thermo Fisher Scientific, Waltham, MA, USA) in an incubator stirrer at 37 °C and diluted to ~10^8^ cfu/mL. Subsequently, 1 mL of standardized bacterial suspensions was added to MAC-T cells, which were previously cultured in a 12-well tissue culture plate (Sarstedt, Nümbrecht, Germany) at 1.2 × 10^5^ cells/mL in Dulbecco’s Modified Eagle Medium supplemented with 10% fetal bovine serum. MAC-T cells and *S. aureus* isolates were co-incubated at 37 °C with 5% CO_2_ for 3 h to enable invasion before gentamicin exposure at 200 mg/mL (Sigma-Aldrich) to kill extracellular bacteria. Plates were incubated for an additional 3 h, and 1 mL of 0.5% Triton X-100 (Sigma-Aldrich) solution was added to release intracellular *S. aureus.* Colony-forming units (cfus) were determined after aliquots of each stage had been serially diluted and spotted on Mueller–Hinton agar (Thermo Fisher Scientific). All plates included wells containing only cells and culture media as sterility controls.

### 2.5. Biofilm-Forming Ability

Biofilms were developed and evaluated using the crystal violet staining method [37]. The 10 *S. aureus* isolates were cultured in tryptic soy broth (TSB; Kasvi, Pinhais, Brazil) supplemented with 1% glucose for 24 h at 37 °C. Then, bacterial suspensions were diluted in a fresh supplemented TSB to 10^7^ cfu/mL. Next, 200 µL of bacteria were inoculated into the wells of a 96-well microplate (Cral, Cotia, Brazil). After 24 h of aerobic incubation, supernatants were removed, and the wells were washed with a 0.9% sterile saline solution. Biofilms were heat-fixed by exposing the plates to hot air at 60 °C for 1 h and stained with 0.5% crystal violet (CV). The dye-bound cells were solubilized with 96% ethanol, and the optical density (OD) was measured at 540 nm using a microtiter-plate reader (Exert Plus UV, Asys Hitech, Eugendorf, Austria). Each plate contained negative controls of sterile TSB (ODNC); moreover, *S. epidermidis* ATCC 12228 and *S. aureus* ATCC 25923 were used as weak and strong biofilm-producing controls, respectively. The scoring criteria were non/weak biofilm-forming if ODNC < OD ≤ 2 × ODNC, moderate if 2 × ODNC < OD ≤ 4 × ODNC, or strong if OD > 4 × ODNC [38].

### 2.6. Minimum Inhibitory Concentration (MIC) and Minimum Bactericidal Concentration (MBC)

For the MIC determination of PHMB NPs, disinfectant procedures were performed as described in our previous study [35], with some modifications regarding the compound concentrations. Moreover, we evaluated the minimum bactericidal concentrations. Briefly, the nanoparticles and disinfectants were prepared in a 96-well microplate (Cral, Cotia, Brazil). The following range of concentrations was obtained in a final volume of 100 µL containing MHB: PHMB NPs and PHMB: 0.01–16 µg/mL. Next, 10 µL of *S. aureus* (n = 10) suspension standardized at 5 × 10^6^ cfu/mL was added to the wells, resulting in a final concentration of ~4.55 × 10^5^ cells/well, and the plates were incubated for 24 h at 37 °C. *S. aureus* ATCC 29213 was also evaluated, and the plates included wells for the control of broth sterility and isolate growth. The MIC was determined as the lowest concentration required to inhibit the growth of bacteria. For MBC assays, 10 µL of the wells that presented concentrations ≥ the MIC were spotted on tryptic soy agar (Kasvi, Pinhais, Brazil) and incubated at 37 °C. After 24 h, the MBC values were determined at the concentration required to kill 99.9% of *S. aureus*.

#### Toxicity to Bovine Mammary Epithelial Cells

The cytotoxicity of PHMB NPs and disinfectants to bovine mammary alveolar cells (MAC-T cells) was evaluated by a colorimetric tetrazolium assay with some modifications [39]. The MAC-T cells were cultured in Dulbecco’s Modified Eagle Medium (Vitrocell–Embriolife) supplemented with 50 µg/mL gentamicin, 25 µg/mL amphotericin B, and 10% fetal bovine serum. Cells (5 × 10^4^ cells/mL) were added to a 96-well tissue culture plate (Kasvi) and incubated at 37 °C with 5% CO_2_. After 24 h, the content of each well was removed, and the cells were washed with a sterile phosphate-buffered saline solution. Adhered cells were incubated with increasing concentrations (between 0.12 and 4 µg/mL) of PHMB NPs and PHMB (Polyorganic Tecnologia, São Paulo, Brazil). Non-treated cells and medium were used as controls. After incubation for 24 and 72 h, the supernatant was discarded, and 0.05% Thiazolyl Blue Tetrazolium Bromide (MTT; Sigma-Aldrich) was applied to the wells. Plates were incubated at 37 °C with 5% CO_2_ for an additional hour. Afterward, MTT was removed, and dimethyl sulfoxide (LabSynth, Diadema, Brazil) was added to solubilize the crystals. The optical density (OD) values were measured at 570 nm using a microplate reader (Spectramax M3, Molecular Devices, San Jose, CA, USA), and the results are expressed as the percentage of cell viability compared to the control wells (100% viability).

### 2.7. Experiment 1: Evaluation of PHMB NP Toxicity and Antibiofilm Activities

#### 2.7.1. Biofilm Inhibition

The ability of PHMB NPs and disinfectants to inhibit biofilm formation by the 10 *S. aureus* isolates was evaluated in 96-well microplates using a modified crystal violet (CV) assay [40]. Briefly, compounds were diluted in TSB media supplemented with 1% glucose, aiming to achieve the final concentrations between 0.12 and 4 µg/mL of PHMB NPs and PHMB. Then, 100 µL of *S. aureus* suspension at 10^7^ cfu/mL was added to the wells, resulting in a final volume of 200 µL. Wells in plates were reserved for broth sterility control (n = 3) and biofilm formation control (n = 3) by isolates. The plates were incubated at 37 °C. After 24 h, the supernatants were discarded, and the wells were washed with a 0.9% sterile saline solution, followed by staining with 0.5% CV for 20 min. The stained biofilms were washed, and the plates were dried for 24 h at room temperature. Subsequently, 200 µL of 96% ethanol was added for resolubilization. Absorbance was measured at 540 nm using a microtiter-plate reader (Asys Hitech, Eugendorf, Austria), and the ability of disinfectants to prevent biofilm formation was measured based on the percentage reduction of biofilm mass compared to the untreated control.

#### 2.7.2. Evaluation of Preformed Biofilm

For biofilm formation, 100 µL of *S. aureus* (n = 10) suspensions at 10^7^ cfu/mL was added to the wells of 2 sterile microplates and incubated for 24 h at 37 °C. Afterward, the medium was removed, and the wells were washed with a 0.9% saline solution. Preformed biofilms were treated with PHMB NPs and PHMB diluted in TSB. After 24 h of incubation, one plate of each isolate was submitted to CV procedures for the evaluation of biofilm mass, considering the OD measurement, as previously described. To determine *S. aureus* viability, preformed biofilms of the other plates were disrupted by the addition of 100 µL of 0.1% Triton X-100 (Sigma-Aldrich) into the wells, followed by sonication for 5 min at 37 °C and 30 kHz. Then, the content of each microplate was serially diluted by homogenizing and transferring 10 µL from the original wells to the wells of a new microplate containing 90 µL of a 0.9% saline solution. The content at 10^−1^ dilution was homogenized, and 10 µL was transferred to the wells of another new microplate to obtain a dilution factor of 10^−2^. These procedures were repeated to achieve a dilution of 10^−7^ using a manual multichannel pipette. Afterward, 10 µL from the wells of the microplates from the dilution factors between 10^−5^ and 10^−7^ were spotted on agar three times [41].

### 2.8. Experiment 2: Evaluation of the Antimicrobial Activity of PHMB NPs on Teat Skin

#### Antimicrobial Activity of PHMB NPs Using the Excised Teat Model

The ability of PHMB NPs and PHMB to reduce bacterial load on teat skin was evaluated by ex vivo assays, with comparisons to conventional disinfectants already used for teat disinfection [42]. *S. aureus* was cultured in a brain–heart infusion broth (Kasvi) and centrifuged at 3000× *g* at 5 °C for 20 min. Pellets were washed and resuspended in phosphate-buffered saline solution before being standardized at 0.5 Mac Farland using a nephelometer (Uniscience, Osasco, Brazil). Then, bacteria were diluted in sterile skimmed milk (Becton, Dickinson, and Co., Franklin Lakes, NJ, USA) to achieve a count of 10^7^ cfu/mL. A total of 120 bovine teats were obtained from a commercial abattoir and selected according to their size and teat end and skin scores. Teats were washed with warm detergent and sterile water, followed by disinfection using 70% ethanol. After drying, two marks were made at 15 and 30 mm from the teat end, and metal clips were used to suspend the teats in a wire. Teat skin was contaminated by immersion in freshly prepared bacterial suspensions until the 15 mm marks. After 5 min, the teats were dipped into 15 mL of PHMB NPs at 167 µg/mL and 0.5% PHMB, 0.5% chlorhexidine digluconate (CHG; Polyorganic Tecnologia, São Paulo, Brazil), 1.4% sodium dichloroisocyanurate (NaDCC; Merck & Co., Darmstadt, Germany), and 0.7% iodine (PVP-I; Sigma-Aldrich) available in the povidone complex up to the 30 mm marks. A total of 120 teats were selected. Considering that 5 compounds (PHMB NPs, PHMB, CHG, NaDCC, and PVP-I) were tested against 10 *S. aureus* isolates and that assays were carried out in duplicate, a total of 100 teats were treated, while 20 teats were used as negative controls. Therefore, for each *S. aureus* challenging solution, 2 teats were treated with each compound, while 2 teats were used as undipped controls. Teats were rinsed with Letheen (Becton, Dickinson, and Co., Franklin Lakes, NJ, USA), with 1% of thiosulfate, and the content was collected in sterile plastic tubes. The rinse contents were plated on Columbia blood agar (Becton, Dickinson, and Co.). The content collected from the undipped controls was serially diluted before plating. After 24 h of incubation at 37 °C, colonies were enumerated by visual analysis, and the results were log10 transformed.

### 2.9. Statistical Analyses

Ex vivo assays were performed in duplicate, while all other assays were conducted in triplicate. The cytotoxicity assay was performed three times, with each run in triplicate. In Experiment 1, the effects of the predictors (PHMB NPs vs. PHMB and concentration levels) on the response variables (cytotoxicity to MAC-T cells, inhibition of biofilm, and preformed biofilm) were initially evaluated using a linear mixed-effects model (LMM). However, the data did not meet the normality assumption for the residuals, even after log-transforming the variables. Consequently, nonparametric statistical methods were employed.

To assess the effect of different concentration levels on cytotoxicity, a Kruskal–Wallis test was conducted to determine whether the medians of the response variables differed across the various concentrations, utilizing the kruskal.test function in R. The Mann–Whitney U test was employed to compare the differences between PHMB NPs and PHMB, as well as assess the effect of time (24 h vs. 72 h) on the medians of the response variables. The wilcox.test function in R was used to compute the test statistics and associated *p*-values for both Mann–Whitney U tests. Statistical analyses were conducted using RStudio (version 2024.09.0+375). Differences were considered significant at *p* < 0.05.

## 3. Results

### 3.1. Nanoparticle Characteristics

The PHMB NPs had an average particle size of 221.13 nm (±11.49), a polydispersity index of 0.15 (±0.01), a count rate of 317.89 kilocounts/s (±15.47), and a zeta potential of 46.55 mV (±1.85).

### 3.2. Antimicrobial Susceptibility Results

The MIC values of 13 antimicrobials were determined to evaluate the *S. aureus* susceptibility patterns (Table 1). The highest frequency of antimicrobial resistance (90%) was observed for amoxicillin and oxytetracycline, followed by penicillin (80%), ampicillin (40%), and tetracycline (30%). In contrast, all isolates were susceptible to ciprofloxacin, enrofloxacin, gentamicin, and lincomycin, and 90% of *S. aureus* were susceptible to cefalexin, cefoxitin, and erythromycin. Cefoxitin was used to evaluate the susceptibility to methicillin, and only isolate 1667 was identified as MRSA. Considering the antimicrobial classes, 90% of isolates were resistant to penicillin and tetracyclines, but all isolates were susceptible to fluoroquinolones, aminoglycosides, and lincosamides. In addition to its resistance to penicillin and tetracyclines, isolate 1658 was resistant to erythromycin (macrolide), while isolates 1667 and 1680 were resistant to cephalosporins; these three isolates were classified as multidrug-resistant (MDR).

### 3.3. Intracellular Infection Results

To evaluate the invasive properties of *S. aureus*, MAC-T cells were infected with five isolates standardized at 10^8^ cfu/mL and exposed to gentamicin to kill extracellular bacteria. After 3 h of gentamicin treatment, extracellular cfu counts were compared to the infective dose. After gentamicin treatment, the *S. aureus* isolate counts were 10^3^ cfu/mL for isolate 1666; 10^4^ cfu/mL for isolates 1650, 1677, and 15AL (reference strain); and 10^5^ cfu/mL for isolates 1651 and 1684. After host cell lysis, intracellular *S. aureus* was released, and the cfu counts were approximately 10^5^ cfu/mL for isolates 1650, 1666, and 1677, and 10^6^ cfu/mL for isolates 1651 and 1684, and 15AL. Increased *S. aureus* counts following MAC-T cell lysis indicate that *S. aureus* isolates were already inside the MAC-T cells and, consequently, protected from gentamicin activity.

### 3.4. Biofilm-Forming Ability Results

The biofilm mass produced by *S. aureus* isolates was measured using the CV assay. Comparing the OD measurements obtained for each isolate to those obtained for TSB media (ODNC = 0.105 ± 0.03), *S. aureus* 1650 (OD of 0.518 ± 0.030), 1651 (0.813 ± 0.119), 1663 (0.928 ± 0.078), 1666 (0.861 ± 0.072), 1677 (0.556 ± 0.030), 1684 (0.566 ± 0.063), and 1688 (0.574 ± 0.014) were classified as strong biofilm formers. On the other hand, isolates 1658 (OD of 0.373 ± 0.005), 1667 (0.360 ± 0.007, and 1680 (0.358 ± 0.025) were classified as moderate biofilm formers.

### 3.5. Minimum Inhibitory Concentration and Minimum Bactericidal Concentration Results

To measure the efficacy of the antimicrobial activity of PHMB NPs and free PHMB against *S. aureus* (n = 10), we determined the MIC and MBC values (Table 2). In relation to PHMB NPs, the range of concentrations required to inhibit *S. aureus* growth varied from 0.25 to 1 µg/mL, and most isolates were inhibited at 0.25 µg/mL. For PHMB, the MIC values were determined at 0.5 µg/mL and 1 µg/mL. In general, the MBC values were determined at greater concentrations compared to the MIC values. The concentrations of PHMB NPs required to kill *S. aureus* were 4 to 16 times greater than the inhibitory concentrations. In general, the bactericidal concentrations of PHMB NPs were lower than those observed for PHMB. The lowest MBC of PHMB NPs was determined at 1 µg/mL for two isolates; on the other hand, 8 µg/mL of PHMB NPs was the highest concentration required and killed one isolate. For most *S. aureus* isolates, the MBC of PHMB was 16 times greater than the MIC values, and eight isolates obtained from mastitis cases were killed at 8 µg/mL.

### 3.6. Experiment 1: Evaluation of PHMB NP Toxicity and Antibiofilm Activities Results

#### 3.6.1. Toxicity to Bovine Mammary Epithelial Cells

**Figure 1:**
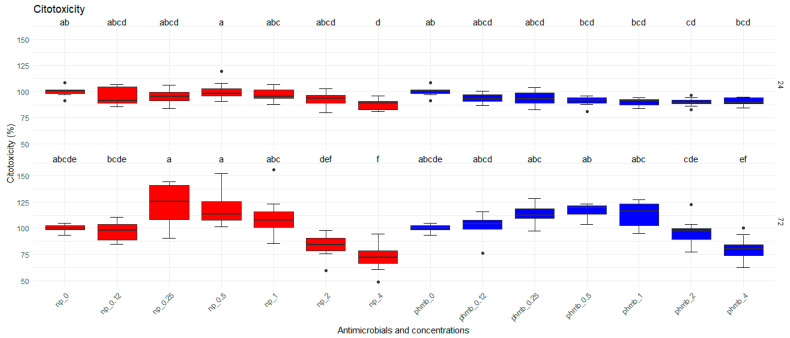
Viability of bovine mammary epithelial cells (MAC-T) after 24 h and 72 h of exposure to increasing concentrations of PHMB NPs and PHMB. The graphs were generated from absorbance measurements obtained from 9 independent replicates. Different letters indicate statistical difference.

Bovine mammary epithelial cells were exposed for 24 h and 72 h to increasing concentrations of PHMB NPs and PHMB for the evaluation of their viability by MTT assays. There was no effect (*p* = 0.862) of PHMB NPs compared to PHMB on the toxicity to bovine mammary epithelial cells; however, significant effects of both concentration (*p* < 0.0001) and time (*p* < 0.0001) were observed on the cytotoxicity to MBC (Figure 1).

#### 3.6.2. Evaluation of PHMB NPs and PHMB on Biofilm Inhibition

**Figure 2:**
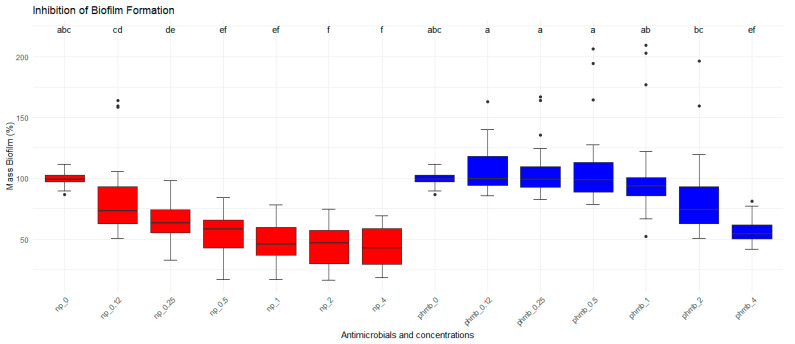
Antibiofilm activities of PHMB NPs and PHMB. PHMB NPs inhibit *Staphylococcus aureus* biofilm formation at lower concentrations than PHMB. Positive controls correspond to 100% of biofilm mass. The graphs were generated from triplicate absorbance measurements. Different letters indicate statistical difference.

The inhibitory effects of PHMB NPs and free PHMB on *S. aureus* biofilm formation were evaluated by measuring the biofilm formed after bacteria were coincubated with different concentrations of both treatments (Figure 2). As controls, untreated biofilms representing 100% of the biofilm mass were used. Nanoparticles significantly reduced the biofilm formation at lower concentrations than PHMB (*p* < 0.05). A concentration effect was also observed (*p* < 0.05) in the two groups. The highest biofilm inhibition for PHMB NPs was observed at 1 µg/mL (53.1%), followed by 2 µg/mL (56%) and 4 µg/mL (56.5%) when compared to the negative control. For PHMB, the highest inhibition was observed at 2 µg/mL (15.8%) and 4 µg/mL (43.3%). At lower PHMB concentrations (≤1 µg/mL), biofilm formation increased compared to the control (0.12 µg/mL: 8.7%; 0.25 µg/mL: 5.5%; 0.5 µg/mL: 6.9%, and 1 µg/mL: 2%, Table 3).

#### 3.6.3. Evaluation of PHMB NPs and PHMB on Preformed Biofilm

**Figure 3:**
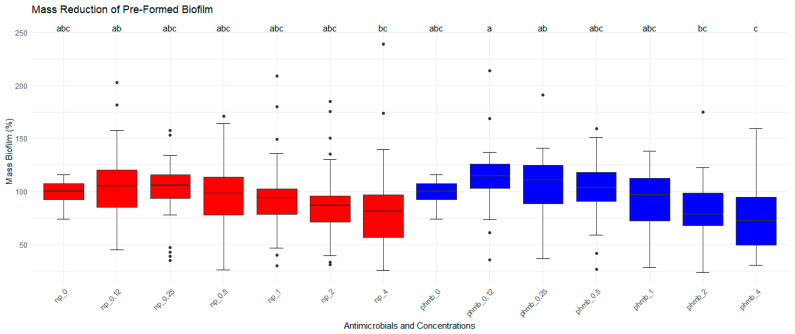
Effects of PHMB NPs and PHMB on the absorbance (measured by optical density-OD) of preformed biofilm for each mastitis-causing *Staphylococcus aureus*. PHMB NPs and PHMB did not eradicate preformed biofilms for most isolates. Positive controls correspond to 100% of biofilm’s mass. The graphs were generated from triplicate absorbance measurements. Different letters indicate statistical difference.

Biofilm formed by *S. aureus* was treated with different concentrations of PHMB NPs and PHMB for the evaluation of their antibiofilm activities. Biofilm mass and bacterial viability were evaluated by CV staining. Untreated biofilms were used as controls, corresponding to 100% of biofilm mass (Figure 3). No significant differences in biofilm mass were observed among PHMB NPs, PHMB, and the control. However, a significant concentration effect was observed for both PHMB NPs and PHMB (*p* < 0.05). At lower concentrations of PHMB and PHMB NPs (0.12 µg/mL and 0.25 µg/mL), an increase in biofilm mass was observed (PHMB: 0.12 µg/mL = 14.5%; 0.25 µg/mL = 6.3%; PHMB NPs: 0.12 µg/mL = 5.4%; 0.25 µg/mL = 1%). Similarly, for both antimicrobials, a reduction in biofilm mass was observed at higher concentrations (PHMB: 2.0 µg/mL = 16.6%; 4.0 µg/mL = 26.1%; PHMB NPs: 4.0 µg/mL = 15.6%, Table 3).

### 3.7. Experiment 2: Evaluation of the Antimicrobial Activity of PHMB NPs on Teat Skin Results

#### Antimicrobial Activity of PHMB NPs and PHMB Using the Excised Teat Model

A total of 120 excised bovine teats had their skin contaminated with *S. aureus* isolates before being exposed to PHMB NPs at 167 µg/mL, 0.5% PHMB, 0.5% CHG, 1.4% NaDCC, and 0.7% PVP-I (except the undipped control teats). Significant differences were obtained regarding the recovered *S. aureus* counts according to the disinfectants used and compared to the undipped control (*p* < 0.05; Table 4). The *S. aureus* counts obtained from the control teats varied from 1.33 log10 cfu/mL (isolate 1651) to 3.45 log10 cfu/mL (isolate 1680). The percentage log10 reduction obtained for PHMB NPs was the lowest and varied from 1.98% to 53.37%. Significant differences (*p* > 0.05) were not observed between the percentage log10 reduction obtained for PHMB NPs and the control; moreover, results obtained for PHMB NPs did not differ from those obtained for PHMB for all tested isolates. The largest frequency of isolates that presented the percentage log10 reduction of 100% compared to undipped teats (*p* < 0.05) was observed for PHMB (five isolates), followed by CHG and NaDCC (four isolates each) and PVP-I (three isolates). On the other hand, isolate 1651 presented the lowest percentage log10 reduction for PHMB (68.32%) and PVP-I (85.34%), while isolate 1666 presented the lowest reduction for NaDCC (73.06%) and CHG (88.12%). In general, the highest values of percentage log10 reductions were observed from teats treated with PVP-I (overall mean of 95.37%), followed by CHG (mean of 94.10%), NaDCC (91.20%), PHMB (90.72%), and PHMB NPs (37.57%).

## 4. Discussion

Due to the growing need to develop new strategies for mastitis treatment and prevention, we developed and tested PHMB NPs against virulent and resistant *S. aureus* and found that PHMB NPs present a high potential to inhibit biofilm formation by *S. aureus*. In the present study, 90% of *S. aureus* (n = 10) were resistant to amoxicillin and oxytetracycline, followed by penicillin G (80%), ampicillin (40%), tetracycline (30%), and ceftiofur (20%). Other studies reported similar patterns of resistance for these antimicrobials [43,44]. The high prevalence of resistance to penicillin and tetracyclines may be related to the common use of these antimicrobial classes in Brazilian dairy herds. Three *S. aureus* isolates were MDR (1658, 1667, and 1680), and this frequency is lower than the frequencies of 46.56% and 57.5% reported by Santos et al. [44] and Brahma et al. [45], respectively. One MDR isolate (1667) was resistant to cefoxitin and classified as MRSA. Despite concerns regarding the severity of diseases caused by MRSA and the risk of zoonotic transmission, the prevalence of MRSA in dairy herds is minimal [46]. Considering the critically important antimicrobials (CIA), all isolates were susceptible to ciprofloxacin and enrofloxacin, while one isolate (10%) was resistant to erythromycin. O’Dea et al. [47] found 0.5% of resistance to erythromycin, and Duse et al. [48] reported a lower frequency of resistance to erythromycin (1.6%) and ciprofloxacin (1.65%). By contrast, Neelam et al. [43] and Kaczorek-Łukowska [49] described that 90.91% and 50% of *S. aureus* were resistant to enrofloxacin, respectively. Compared to other studies performed in Brazil, Santos et al. [44] reported *S. aureus* resistance to enrofloxacin and erythromycin (3.85%), while Mesquita et al. [3] found higher frequencies of resistance to ciprofloxacin (9%) and erythromycin (17%). Although all studies were performed in Southeast Brazil, the exposure of *S. aureus* to different antimicrobial protocols may explain these differences. Even though fluoroquinolones are widely used for mastitis treatment in Brazil [34], the Food and Drug Administration (FDA) prohibited the use of fluoroquinolones in food-producing animals in the United States in 1997 due to risks to human health and AMR. Nonetheless, the use of CIA should be avoided or eliminated in dairy herds, since they do not provide any higher efficacy against mastitis pathogens compared to non-CIA [50].

Invasive and biofilm-forming strains of *S. aureus* are protected from cows’ immune systems and antimicrobials, which may partially explain why *S. aureus* is often associated with therapy failures and chronic mastitis [51]. We selected five *S. aureus* isolates, and all of them invaded MAC-T cells. *S. aureus*’s ability to invade bovine mammary epithelial cells has been reported by previous studies [14,52]. Even though all selected isolates invaded MAC-T cells, the mechanisms of interaction with bovine mammary epithelial cells and immune cells, as well as the time required for invasion and apoptosis induction, depend on the strains and the AMR profile [53,54]. Interestingly, all isolates tested in the invasion assay were resistant to penicillin, and only isolate 1650 was susceptible to tetracyclines. As mentioned, these antimicrobial classes are commonly used for mastitis treatment in Brazilian dairy herds [55], although further analyses should be performed for a better establishment of this association.

Biofilm formation involves cell attachment and the production of an extracellular matrix, and some genes are involved in this process, especially icaA and icaD. Moreover, the biofilm-associated protein (bap) encodes the bap surface protein, which is involved in the primary attachment to inert and biological surfaces in addition to intercellular adhesion [56]. In this study, most isolates (n = 7) were strong biofilm formers, and the MDR isolates (1658, 1667, and 1680) were classified as moderate biofilm formers. Lower frequencies of moderate and strong biofilm formers were reported by Thiran et al. [56] and Ren et al. [57]. However, our results are similar to Costa Krewer et al. [58], who reported 72.5% of strong biofilm formers, and Marques et al. [59], who found 30% of moderate formers. Herein, all *S. aureus* isolates were moderate or strong biofilm formers and were resistant to antimicrobials used for mastitis treatment, especially penicillin and tetracyclines. This finding is in accordance with da Costa Krewer et al. [58], who reported that 74% of strong biofilm-forming *S. aureus* were resistant to beta-lactam antimicrobials and tetracyclines. Moreover, the MDR and MRSA isolate 1667 was a moderate biofilm former, and Thiran et al. [56] classified MRSA isolates as moderate and strong biofilm formers. Bacteria inside the biofilm tolerate antimicrobial concentrations 1000 times greater than planktonic bacteria, and physiological alterations of bacteria inside biofilms and reduced penetration of antimicrobials may explain biofilm antimicrobial resistance [60]. Moreover, the horizontal transfer of antimicrobial resistance genes is potentially easier for these bacterial communities because of their proximity [61]. Therefore, our findings suggest the need for improvements in control practices in order to reduce or eliminate these isolates in dairy herds.

The MIC values of PHMB NPs and PHMB reflect their bacteriostatic activity, while the MBC values reflect their bactericidal activity. In addition, the MIC can reflect bactericidal activity that would be confirmed by the MBC. Most *S. aureus* isolates were inhibited at 0.25 µg/mL, and half were killed at 4 µg/mL of PHMB NPs, while greater concentrations of PHMB were required to inhibit (0.5 µg/mL) and kill (8 µg/mL) most isolates. Compared to our previous study ([28]), no differences were observed between the MIC values (0.5 µg/mL) of PHMB required to inhibit most *S. aureus* isolates; however, PHMB NPs inhibited most *S. aureus* isolates at lower values (0.03 µg/mL). This finding may be explained by the PHMB NP composition, which included natural polymers (alginate and chitosan). Synthetic polymers (such as PHMB) usually present higher purity and homogeneity than natural polymers. However, PHMB NPs also present alginate and chitosan in their formulation, whose properties can be affected because of the batch variability within the production and purification processes, compromising the reproducibility of NP synthesis [62].

Polymeric nanoparticles have been studied against mastitis pathogens because of their biocompatibility, biodegradability, low toxicity, and antimicrobial properties. Recently, Orellano et al. [63] synthesized three chitosan NPs under different conditions. They found that the smallest (134 nm) chitosan NP 1 presented the highest antimicrobial activity against *S. aureus* (MIC = 200 µg/mL; MBC = 400 µg/mL). On the other hand, chitosan NPs 2 (234 nm) and 3 (243 nm) inhibited and killed *S. aureus* at the same concentrations (MIC = MBC = 800 µg/mL). Polypyrrole NPs were developed by Acosta et al. [64], and their antimicrobial activity was evaluated against mastitis-causing *S. aureus* obtained from cows (81.2% of tested isolates) and goats. No differences were observed between the MIC values (125 µg/mL) required to inhibit *S. aureus* obtained from cows or goats. Thus, *S. aureus* growth was inhibited at higher concentrations of polypyrrole NPs than PHMB NPs or PHMB. Alginate has also been used to enhance the antimicrobial activity of antimicrobials. Sodium alginate-stabilized antibiotics and NPs were tested against mastitis-causing *Streptococcus agalactiae* and *Klebsiella pneumoniae* [65]. Tylosin and MgO NPs presented higher antimicrobial activities (an MIC of 9.76 µg/mL for *K. pneumoniae* and 26.04 µg/mL for *S. agalactiae*) than ampicillin and MgO NPs (an MIC of 26.04 µg/mL for *K. pneumoniae* and 78.13 µg/mL for *S. agalactiae*). Alginate/chitosan systems have been used for biomedical applications because when they are mixed, they form a stable polyelectrolyte complex and work synergistically to protect the active compounds from oxidation, hydrolysis, and enzymatic degradation in nanoformulation [66]. However, the interactions among chitosan, alginate, and PHMB that may explain the antimicrobial activity of PHMB NPs remain unclear, and further studies are necessary for a better understanding.

### 4.1. Experiment 1: Evaluation of PHMB NP Toxicity and Antibiofilm Activities

The significant burden of antimicrobial-resistant infections on public health leads to concerns and pressures regarding antimicrobial use and residues in animal-based foods and cross-contamination, resulting in investigations of non-antibiotic alternatives for livestock farming [67]. With a view toward affordable and safe options for mastitis control, PHMB NPs were evaluated considering their potential use for intramammary treatment. As previously mentioned, chitosan/alginate systems present ideal features for drug delivery. Moreover, chitosan has been widely studied for tissue regeneration and skin wound repair because of its ability to promote hemostasis and proliferation of granulation, in addition to its anti-inflammatory properties [68].

Bovine mammary epithelial cells cannot be compromised by the treatments administered via the intramammary route. The mechanisms involved in PHMB NP toxicity to MAC-T cells have not been studied, but NPs usually cause cell injury and death due to high oxidative stress and alterations in intracellular calcium homeostasis [69]. Herein, the effects of PHMB NPs on cell viability were dose-dependent, but the concentration required to reduce cell viability was four times higher than the inhibitory concentrations for *S. aureus* (maximum of 1 µg/mL). Consequently, PHMB NPs’ antimicrobial properties occur at lower concentrations than the toxic concentrations for MAC-T cells. On the other hand, PHMB reduced around 10% of cell viability at concentrations ≥ 1 µg/mL after 24 h of incubation, but PHMB did not alter cell viability over a longer period (72 h). PHMB is considered a safe polymer. In exposed mammalian cells, it enters and localizes within endosomes excluded from nuclei; this localization pattern is consistent with its low toxicity properties in vivo [27]. Moreover, PHMB enters MAC-T cells and acts selectively against intracellular *S. aureus,* killing up to 99.9% of them [14]. Thus, PHMB may also be considered safe for intramammary application, provided concerns about possible residues in foods can be addressed.

New strategies for mastitis treatment should be safe for host cells and focus on the virulence properties of bacteria. Biofilms are often associated with AMR and chronic infections, and nanotechnology represents a novel approach to prevent biofilm formation and eradicate preformed biofilms [70,71]. We evaluated the ability of PHMB NPs and PHMB to prevent biofilm formation by *S. aureus* and found that PHMB NPs significantly reduced biofilm formation at concentrations between 1 µg/mL and 4 µg/mL, on average. The biofilm formation obtained at these concentrations was significantly lower than that obtained for PHMB, which did not achieve > 50% biofilm inhibition at any tested concentration. This result indicates the higher efficacy of the nanoformulation in preventing biofilm formation compared to the bulk compound. Studies reported that MRSA isolates present a higher prevalence of virulence genes than methicillin-sensitive *S. aureus*, and the *icaA* and *icaD* genes were closely associated with the *mecA* gene [72,73]. Although biofilm formation presents a positive correlation with the presence of the *mecA* gene, only one isolate (1667) was classified as MRSA due to cefoxitin resistance; this isolate was also categorized as a moderate biofilm former. In general, biofilm formation was reduced at concentrations higher than the MIC obtained for PHMB NPs (between 0.25 µg/mL and 1 µg/mL) and PHMB (0.5 µg/mL to 1 µg/mL). In relation to other polymeric NPs, significant inhibitory effects on biofilm formation were reported at higher concentrations than those obtained in this study. Aguayo et al. [74] found that sub-MIC doses of chitosan NPs significantly inhibited biofilm formation by mastitis-causing *Pseudomonas* spp. at concentrations ≥70 µg/mL. Orellano et al. [63] reported that the smallest chitosan NPs that they synthesized reduced 80% of biofilm formation by one *S. aureus* isolate at 800 µg/mL. The inhibition of biofilm formation by NPs seems to be associated with the interaction between NPs and planktonic bacteria, acting on the mechanisms related to bacterial adhesion to living tissues and surfaces [75].

On average, isolates increased their biofilm formation at low concentrations of PHMB NPs and PHMB. For PHMB NPs, concentrations ≥ the MIC (0.5 µg/mL) effectively reduced biofilm formation, while PHMB required concentrations > the MIC. Subinhibitory concentrations of silver-based NPs also increased the biofilm formation of *Pseudomonas aeruginosa* and *S. epidermidis* [76,77], and zinc NPs increased biofilm formation by *P. putida* [78]. Sub-MIC doses of chlorhexidine, which is a biguanide like PHMB, induced the biofilm formation of MRSA and mastitis-causing *S. aureus* and *Streptococcus agalactiae* [79,80]. Considering that some bacterial strains are more resistant to some antimicrobials, bacterial cells can adapt to low concentrations of antimicrobials and form biofilms to protect themselves [81]. Consequently, the use of antimicrobials at lower doses than those required for optimal antimicrobial activity, which is determined by MIC and MBC values, can be ineffective and cause negative consequences, such as AMR and biofilm formation [82].

The antibiofilm activities of PHMB NPs and PHMB against preformed biofilms were also evaluated after 24 h of treatment. Compared to untreated controls, PHMB NPs and PHMB did not significantly reduce biofilm mass at lower concentrations (0.12 µg/mL to 0.5 µg/mL). Similarly, Nguyen et al. [83] also reported that α-mangostin polymeric NPs were less effective against preformed biofilms. Although it is not possible to establish a direct comparison regarding NP concentration, 48 mmol/L of α-mangostin polymeric NPs was necessary to reduce 27% of the biofilm mass of one *S. aureus* standard strain and 22% of the biomass previously formed by one MRSA. Herein, we found that PHMB NPs at lower concentrations (0.5 µg/mL and 1 µg/mL) reduced 3% and 3.6% of biofilm mass formation, while higher concentrations (2 µg/mL and 4 µg/mL) reduced 10.4% and 15.6% of the biofilm mass formed. On the other hand, Xu et al. [84] reported that cinnamaldehyde–chitosan NPs disrupted 48.10% of *S. aureus* preformed biofilms after repeated treatment for 2 days.

Some factors are involved in NPs’ efficacy against preformed biofilms, such as their charge and size. Generally, positively charged NPs, such as chitosan NPs, penetrate biofilms more easily due to their higher interaction with negatively charged biofilm structures [85]. As PHMB NPs present the external layer composed of chitosan, this criterion is fulfilled. It has also been reported that the ideal size of NPs to penetrate biofilm structures is between 5 nm and 200 nm and should not exceed 500 nm [86]. Cinnamaldehyde–chitosan NPs synthesized by Xu et al. [84] presented around 298.1 nm, while PHMB NPs measured around 221.13 nm. As the treatment with cinnamaldehyde–chitosan NPs was repeated for 2 days in preformed biofilms, maybe PHMB NPs also need an extended treatment duration to reduce biofilm mass in a more effective way. Thus, more investigations are necessary for a better understanding of PHMB NP activity against preformed biofilms.

### 4.2. Experiment 2: Evaluation of the Antimicrobial Activity of PHMB NPs on Teat Skin

Post-milking teat disinfection is one of the main practices recommended to prevent IMI caused by *S. aureus*. Aiming to develop a novel and affordable approach for *S. aureus* control in dairy herds, we evaluated the efficacy of PHMB NPs using the excised teat model. In addition to the active ingredient, our PHMB NPs present natural polymers (alginate and chitosan) in their composition. Polymeric NPs are used for topical delivery since they can improve skin permeation and provide a controlled and sustained drug release [87]. Moreover, chitosan and alginate also present mucoadhesive and bioadhesive properties [66] that may form a protective film against bacterial invasion between milkings.

In this experiment, we used the excised teat model to evaluate the antimicrobial activity of PHMB NPs and PHMB as an initial screening test since this model is affordable and practical. Nevertheless, this assay is not suitable for measuring other important properties of teat disinfectants, such as the healing of teat lesions, prevention of teat chapping, the persistence of antimicrobial activity on teat skin, and the disinfectant’s ability to prevent IMI in cows [88]. This is the first study that has evaluated the potential for NPs to reduce *S. aureus* loads on teat skin; however, contrary to our hypothesis, PHMB NPs presented the lowest reduction in *S. aureus* load. The percentage log10 reduction obtained from teats treated with PHMB NPs (mean of 37.57%) did not differ from those obtained for PHMB (mean of 90.72%) and the other disinfectants. Furthermore, the reduction in *S. aureus* counts on teats treated with PHMB NPs did not differ from the control teats, but this lack of significant differences between treated and control teats was also observed for the other disinfectants, depending on the *S. aureus* isolate.

The concentration of the active compound, the contact time, and the bacteria are the main factors that affect the efficacy of disinfectants [89]. The final concentration of PHMB in this nanoformulation was 167 µg/mL, which is almost 30 times lower than the concentration tested for free PHMB (0.5% or 5000 µg/mL); even so, no significant differences were obtained between their antimicrobial activity. Nevertheless, polymeric NPs usually control the release of the active compound, enhancing their therapeutic properties over time [90]. Thus, the PHMB NP contact time of 10 min was not sufficient to observe activity, and this was a limitation of this experiment, in addition to the fact that the concentration of PHMB in the nanoformulation (167 µg/mL or 0.0167%) was much lower than the concentrations of the free compounds. This difference may compromise a fair comparison of their antimicrobial activities.

Moreover, we observed that some isolates were not susceptible to certain disinfectants already used in dairy herds. Although a direct comparison with our study is not possible, Schwenker et al. [91] determined the MIC values of 0.215% chlorhexidine and 3.5% lactic acid to inhibit mastitis pathogens from the genera *Staphylococcus*, *Streptococcus*, and *Corynebacterium*. The authors reported an increase in MIC values obtained for lactic acid against all tested isolates after an intervention of 6 days using these disinfectants for dipping. Our results, as well as those obtained by Schwenker et al. [91], may be related to bacteria exposure and adaptation to these disinfectants used in dairy herds since the consistent use of disinfectants can lead to reduced susceptibility of some pathogens [92].

Few studies have reported the efficacy of novel compounds on the reduction of bacterial load using ex vivo or in vivo assays. Klostermann et al. [93] developed an alternative formulation composed of the living *Lactococcus lactis* DPC 3251, which produces a bacteriocin called Lacticin. Using in vivo experiments, teats were exposed to challenging suspensions of mastitis-causing pathogens. After a contact time of 10 min, the percentage log10 reduction obtained for *S. aureus* was 80%, and it achieved 97% for *S. dysgalactiae*. At the same contact time, the antimicrobial activity of Lacticin [46] was higher than that obtained for PHMB NPs. Moreover, the Lacticin [46] activity was retained at 53% of its original level after storage for three weeks, while PHMB NPs also presented their measurements as stable after one month of storage.

Enger et al. [42] also used the excised teat model to evaluate the efficacy of four dip products against eleven bacterial species isolated from mastitis. Four *S. aureus* strains were tested, and the lowest percentage log10 reduction was obtained for 1% H_2_O_2_ (56.9%), followed by 1% chlorine dioxide (89.4%) and 0.5% iodophor (88.5%). These results are lower than the mean observed for the disinfectants used (except PHMB NPs) in the present study. The percentage log10 reduction of the least effective disinfectant (1% H_2_O_2_) was greater than that obtained for PHMB NPs. In contrast, the highest antimicrobial activity (100% reduction) was observed for 1% iodophor. This result is also greater than that obtained for PVP-I by us, despite the differences related to the concentration of the active compound.

The ability of 96 commercially available teat disinfectants to reduce bacterial load on teat skin was also evaluated by Fitzpatrick et al. [94]. The dip solutions were composed of nine active compounds, combined or not, at various concentrations. The lowest percentage log10 reduction (39%) of *Staphylococcus* spp. was observed for chlorine dioxide products, which was similar to that obtained for PHMB NPs and lower than our results for NaDCC. On the other hand, staphylococcal isolates were more susceptible to products composed of diamine (94.7%) or 2% lactic acid combined with 0.6% chlorhexidine (100% reduction). Among the iodine-based products, the highest reduction observed for *Staphylococcus* spp. was 78.1%, which is lower than that observed in our PVP-I study. Moreover, the authors reported that CHG was the most common active ingredient, and the percentage of log10 reduction of 82.5% obtained for the formulation with 0.5% CHG was lower than that obtained for PHMB NPs and CHG in this study. In general, the results of percentage log10 reduction obtained in our study for the free disinfectants were greater than those reported by other studies, and this may be explained because commercial formulations contain additional ingredients, such as emollients and surfactants, that might interfere with antimicrobial activity [95].

## 5. Conclusions

In this study, we observed using in vitro assays that PHMB NPs and PHMB were safe for bovine mammary epithelial cells and that PHMB NPs were more effective in preventing biofilm formation by virulent *S. aureus*; however, both compounds presented limited activity against preformed biofilms. Moreover, PHMB NPs presented the lowest reduction in *S. aureus* load on teat skin compared to PHMB and other disinfectants. We suggest further investigations related to the release rate and time–kill curve of PHMB NPs for a better understanding of their mode of action and effects on teat skin over time, considering their usage for teat disinfection.

## Figures and Tables

**Table 1:** Minimum inhibitory concentrations (µg/mL) of antimicrobials against 10 mastitis-causing *Staphylococcus aureus* isolates and relative frequency of resistance ^1^.

Antimicrobial	Tested Isolate										Resistance (%)
	1650	1651	1658	1663	1666	1667	1677	1680	1684	1688	
Amoxicillin	0.25	1	0.25	0.25	0.25	0.25	0.25	0.25	0.25	0.06	90
Ampicillin	0.25	0.5	0.12	0.12	0.12	0.25	0.25	0.12	0.12	0.06	40
Cefalexin	0.5	1	2	2	2	8	2	1	2	0.5	10
Cefoxitin	2	2	2	2	2	8	2	2	2	2	10
Ceftiofur	1	2	2	2	2	8	2	4	2	1	20
Ciprofloxacin	0.06	0.12	0.25	0.12	0.12	0.12	0.12	0.12	0.12	0.06	0
Enrofloxacin	0.12	0.06	0.12	0.12	0.06	0.12	0.06	0.12	0.06	0.06	0
Erythromycin	0.12	0.12	4	1	1	1	0.5	1	1	1	10
Gentamicin	0.25	0.25	0.12	1	0.5	0.25	0.25	0.25	0.25	0.25	0
Lincomycin	0.12	0.25	0.25	0.12	0.25	0.12	0.12	0.25	0.12	0.12	0
Oxytetracycline	1	8	8	8	8	4	4	4	8	4	90
Penicillin G	0.25	0.25	0.5	0.25	0.03	0.5	0.25	0.25	0.25	0.03	80
Tetracycline	1	1	1	4	1	4	1	1	64	1	30

^1^ *S. aureus* was classified as susceptible or resistant to antimicrobials according to the European Committee on Antimicrobial Susceptibility Testing clinical breakpoints.

**Table 2:** Concentrations of PHMB NPs and PHMB required to inhibit growth and kill mastitis-causing *Staphylococcus aureus* isolates using in vitro assays.

Tested Isolate	PHMB NPs ^1^		PHMB ^2^	
	MIC ^3^	MBC ^4^	MIC ^3^	MBC ^4^
1650	0.25	2	0.5	2
1651	0.25	1	0.5	8
1658	0.5	4	0.5	8
1663	0.25	4	0.5	4
1666	0.25	2	1	8
1667	0.25	4	0.5	8
1677	0.25	1	1	8
1680	0.5	4	0.5	8
1684	1	8	0.5	8
1688	0.5	4	0.5	8
ATCC 29213 ^5^	0.5	4	0.5	8

^1^ PHMB NPs—Polyhexamethylene biguanide nanoparticles. ^2^ PHMB—Polyhexamethylene biguanide. ^3^ Minimum inhibitory concentration (µg/mL). ^4^ Minimum bactericidal concentration (µg/mL). ^5^ Concentrations (µg/mL) obtained for the *Staphylococcus aureus* American Type Culture Collection 29213.

**Table 3:** Percentage of preformed *Staphylococcus aureus* biofilm mass and inhibition of *Staphylococcus aureus* biofilm following treatment with PHMB and PHMB NPs at various concentrations.

Concentration	Preformed Biofilm		Biofilm Inhibition	
	Treatments			
	PHMB NPs	PHMB	PHMB NPs	PHMB
0	100 ^1^	100	100	100
0.12	105.3	114.5	82.9	108.6
0.25	101	106.2	64.9	105.5
0.5	97	100.9	53.6	106.9
1	96.3	91.2	46.8	102
2	89.6	83.4	43.9	84.2
4	84.4	73.9	43.5	56.7

^1^ Values were calculated in comparison with untreated biofilm controls, corresponding to 100% of biofilm mass.

**Table 4:** *Staphylococcus aureus* recovered from teat skin and mean percentage log reduction obtained for each disinfectant compared to undipped control teats.

Tested Isolate	Control (Log) ^1^	PHMB NPs ^1^		PHMB ^1^		CHG ^1^		NaDCC ^1^		PVP-I ^1^	
		Log ^2^	% Log Reduction ^3^	Log ^2^	% Log Reduction ^3^	Log ^2^	% Log Reduction ^3^	Log ^2^	% Log Reduction ^3^	Log ^2^	% Log Reduction ^3^
1650	2.60 ^A^	1.76	32.27 ^AB^	0.15	94.21 ^B^	0.23	91.32 ^B^	0.56	78.32 ^AB^	0	100 ^B^
1651	1.33 ^A^	1.30	1.98 ^AB^	0.42	68.32 ^AB^	0	100 ^B^	0	100 ^B^	0.19	85.34 ^AB^
1658	2.79 ^A^	1.30	53.37 ^AB^	0	100 ^B^	0.24	91.45 ^B^	0	100 ^B^	0.12	95.72 ^B^
1663	2.58 ^A^	1.76	31.49 ^AB^	0.33	87.37 ^AB^	0	100 ^B^	0	100 ^B^	0.19	92.45 ^AB^
1666	2.64 ^A^	1.54	41.77 ^AB^	0.46	82.42 ^B^	0.31	88.12 ^B^	0.71	73.06 ^AB^	0.33	87.68 ^B^
1667	2.88 ^A^	1.81	37.05 ^AB^	0	100 ^B^	0	100 ^B^	0.37	87.19 ^B^	0	100 ^B^
1677	2.90 ^A^	1.62	44.10 ^AB^	0	100 ^B^	0.08	97.40 ^B^	0	100 ^B^	0.08	97.40 ^B^
1680	3.45 ^A^	1.77	48.70 ^AB^	0	100 ^B^	0	100 ^B^	0.12	96.55 ^B^	0.08	97.82 ^B^
1684	2.76 ^A^	1.39	49.58 ^AB^	0.69	74.84 ^AB^	0.67	75.72 ^AB^	0.42	84.78 ^AB^	0.08	97.27 ^B^
1688	2.47 ^A^	1.60	35.35 ^AB^	0	100 ^B^	0.08	96.96 ^B^	0.19	92.13 ^B^	0	100 ^B^

^A–B^ Percentage log_10_ reductions that do not present the same superscript in the same row were significantly different (*p* < 0.05). ^1^ Disinfectants: PHMB NPs—Polyhexamethylene biguanide nanoparticles (167 µg/mL); PHMB—Free polyhexamethylene biguanide (0.5%); CHG—Chlorhexidine digluconate (0.5%); NaDCC—Sodium dichloroisocyanurate (1.4%); PVP-I—Povidone iodine (0.7%). ^2^ Log_10_ of colony-forming units/mL of recovered *S. aureus* isolates (*n* = 10) from control or treated teats. ^3^ Percentage of log_10_ cfu recovered from teats treated by disinfectants compared to undipped teats.

## Data Availability

The data are contained within this manuscript.

## Abbreviations

The following abbreviations are used in this manuscript:

AMR	Antimicrobial resistance
ATCC	American Type Culture Collection
CHG	Chlorhexidine digluconate
CIA	Critically important antimicrobials
CV	Crystal violet
DLS	Dynamic light scattering
EUCAST	European Committee on Antimicrobial Susceptibility Testing
IMI	Intramammary infections
LMM	Linear mixed-effects model
MAC-T cells	Bovine mammary epithelial cells
MALDI-TOF MS	Matrix-assisted laser desorption/ionization time-of-flight
MBC	Minimum bactericidal concentration
MDR	Multidrug-resistant
MG	Mammary gland
MHB	Mueller–Hinton broth
MIC	Minimal inhibitory concentrations
MRSA	Methicillin-resistant *Staphylococcus aureus*
MTT	Thiazolyl Blue Tetrazolium Bromide
NaDCC	Sodium dichloroisocyanurate
NPs	Nanoparticles
OD	Optical density
ODNC	Optical density of negative control
PHMB	Polyhexamethylene biguanide
PVP-I	Povidone iodine
TSB	Tryptic soy broth
